# Structural Identification and Molecular Interaction Modeling Analysis of Antioxidant Activity Selenium-Enriched Peptides from Selenium-Enriched *Pleurotus eryngii*

**DOI:** 10.3390/antiox14050586

**Published:** 2025-05-13

**Authors:** Lili Chen, Menghan Nie, Jing Yang, Weibin Zhang, Tom Hsiang, Yuji Jiang, Baogui Xie, Bingzhi Chen

**Affiliations:** 1 College of Food Science, Fujian Agriculture and Forestry University, Fuzhou 350002, China; llchen04@163.com (L.C.); jyj1209@163.com (Y.J.); 2 Mycological Research Center, Fujian Agriculture and Forestry University, Fuzhou 350002, China; 3 School of Environmental Sciences, University of Guelph, Guelph, ON N1G 2W1, Canada; 4 Key Laboratory of Subtropical Characteristic Fruits, Vegetables and Edible Fungi Processing (Co-Construction by Ministry and Province), Ministry of Agriculture and Rural Affairs, Fuzhou 350002, China

**Keywords:** SePEP, antioxidant activity, structural identification, molecular interaction modeling

## Abstract

This study investigated the structure–activity relationships between SePEPs (selenium-enriched peptides) and PEPs (selenium-free peptides) and compared the antioxidant activities of SePEPs and PEPs. The results showed that SePEPs exhibited higher antioxidant activity than PEPs at the same molecular weight, with the molecular weights of 0–3500 Da exhibiting the highest in vitro antioxidant activity. Chelation between selenium and peptides led to a more compact structure and increased particle density in SePEPs. A spectroscopic analysis revealed new peaks and redshifts in SePEPs, along with a higher content of hydrophobic amino acids than PEPs. A molecular interaction modeling analysis indicated that hydrogen bonding and hydrophobic interactions primarily drove the binding between selenium-containing peptides and 1,1-diphenyl-2-picrylhydrazyl (DPPH). Moreover, the solid-phase synthesized MSePGP exhibited significantly greater antioxidant activity than glutathione at high concentrations. At 10 mg/mL, the DPPH radical scavenging rate of MSePGP was 68.5 ± 2.2%. These findings provide a theoretical basis for the design and synthesis of selenium-enriched peptides with enhanced antioxidant properties.

## 1. Introduction

Selenium is an essential trace element in the human body, playing a vital role in various physiological processes such as oxidative stress, immune function, and thyroid health [1]. Many studies have shown that selenium-enriched proteins have strong antioxidant activity [2,3]. Selenium deficiency is associated with numerous health issues, including *Keshan disease*, thyroid disorders, and cancer [4]. However, excessive selenium intake can lead to selenium toxicity, which can cause skin damage, hair loss, neurological disorders, and even death [5].

Selenium mainly exists in two forms in nature: inorganic selenium and organic selenium. Inorganic selenium has a certain toxicity, whereas organic selenium has a certain biological activity and can be obtained through biotransformation [6,7]. *Pleurotus eryngii* is rich in a variety of phytochemicals, including polyphenols, sterols, coumarins, triterpenoids, and carotenoids, all of which contribute to its diverse biological activities, such as antioxidant, anti-inflammatory, and antitumor effects. In addition to these compounds, *P. eryngii* also contains proteins and polysaccharides. Notably, it can absorb inorganic selenium from the cultivation substrate and convert it into organic selenium compounds, such as selenium-enriched proteins and selenium-enriched polysaccharides, thereby achieving effective selenium biofortification [8,9]. Furthermore, selenium biofortification has been shown to significantly enhance both the total selenium content and the antioxidant activity of *P. eryngii* [10,11].

Previous studies have shown that selenium-enriched peptides can be extracted from selenium-enriched plants such as green beans [12] and millet [13]. Selenium-enriched peptides possess higher biological activity than their non-selenium counterparts and can serve as an effective source of selenium for humans [14,15].

LC-MS/MS offers significant advantages in the discovery of new peptide sequences, identification of modified peptides, and detection of amino acids, enabling the determination of peptide molecular weight and amino acid sequences [16,17]. Molecular interaction modeling, based on the “lock-and-key” principle of ligand–receptor interactions, is a technique used to simulate the interaction between small ligand molecules and receptor biomolecules. This approach can be widely applied to study the interaction of selenium-enriched peptides with 2,2-diphenyl-1-picrylhydrazyl (DPPH), and it plays a crucial role in the research, design, and synthesis of antioxidant drugs [18]. At present, many bioactive peptides such as umami peptides [19] and immunomodulatory peptides [20] have been discovered by molecular interaction modeling technology, and their conformational relationship has been analyzed.

This study aimed to extract selenium-enriched proteins from selenium-enriched *P. eryngii* through alkali extraction and acid precipitation, followed by enzymatic hydrolysis to obtain selenium-enriched peptides. After isolation and purification, the strongest antioxidant activity fractions were screened for structural characterization. LC-MS/MS was used to identify selenium-containing peptide sequences, and molecular interaction modeling was employed to analyze the binding sites of selenium on the peptides and their interaction with DPPH. The results provided a theoretical basis for analyzing the structure of selenium-enriched peptides and synthesizing selenium-enriched peptides with higher antioxidant activity.

## 2. Materials and Methods

### 2.1. Materials and Reagents

Selenium-enriched *P. eryngii* was cultivated by Zhangzhou Tianhua Edible Mushroom Co., Ltd. (Zhangzhou, Fujian, China), and the selenium content in fresh fruitbodies of selenium-enriched *P. eryngii* was 14.65 mg/kg. Sodium chloride (NaCl), methanol, and acetic acid were purchased from Xilong Scientific Co., Ltd. (Shantou, Guangdong, China). The pre-stained protein marker was purchased from Chengdu Zhengneng Biotechnology Co., Ltd. (Chengdu, Sichuan, China). Acetonitrile was obtained from Thermo Fisher Scientific Co., Ltd. (Waltham, MA, USA). The dithiothreitol (DTT) solution, iodoacetamide (IAM) solution, and other reagents were purchased from Sigma-Aldrich Co. Ltd. (Shanghai, China).

### 2.2. Preparation of SePEPs

Selenium-enriched peptides (SePEPs) were prepared following previous methods [21]. Briefly, selenium-enriched protein powder was mixed with distilled water at a material-to-liquid ratio of 1:20 (*w*/*v*) and preheated in a water bath at 75 °C for 30 min. The pH of the mixture was then adjusted according to the optimal conditions for enzymatic hydrolysis. Protease was added, and the mixture was incubated in a constant-temperature water bath under specific enzymatic digestion conditions. Upon completion of hydrolysis, the reaction was terminated by heating at 95 °C for 15 min to inactivate the enzyme. After cooling to room temperature, the solution was centrifuged, and the supernatant was collected.

The obtained hydrolysate was subjected to molecular weight fractionation using dialysis bags with molecular weight cut-offs of 3500 Da and 8000 Da, resulting in two peptide fractions: 0–3500 Da and 3500–8000 Da. These fractions were concentrated and freeze-dried to obtain SePEP powders. Selenium-free peptides (PEPs) were prepared using the same procedure, starting with non-selenium-enriched protein powder.

### 2.3. Determination of Antioxidant Activity of SePEPs and PEPs In Vitro

The antioxidant activities of SePEP and PEP fractions with molecular weights of 0–3500 Da and 3500–8000 Da were determined using the Solarbio antioxidant assay kit (Beijing, China) based on the methods described by Chen et al. [21]. The assays included DPPH radical scavenging, 2,2′-casino-bis (3-ethylbenzothiazoline-6-sulfonic acid) (ABTS) radical scavenging, hydroxyl radical scavenging, and ferric reducing/antioxidant power (FRAP). The SePEP and PEP fractions with the strongest in vitro antioxidant activity were selected for structural identification.

### 2.4. Transmission Electron Microscopy (TEM)

The SePEP and PEP samples were placed on a 400-mesh copper grid with a carbon film. The grids were dried using an infrared lamp and then examined using an HT7800 transmission electron microscope (Hitachi Scientific Instruments Ltd., Tokyo, Japan) to observe the microstructures of SePEPs and PEPs.

### 2.5. UV Absorption Spectra

The SePEP and PEP samples were dissolved in distilled water to 0.25 mg/mL, and distilled water was used as a control. UV spectra were measured using an Evolution Pro UV-visible spectrophotometer (Thermo Fisher Scientific, Waltham, MA, USA) in the wavelength range of 190–400 nm with 1 nm intervals.

### 2.6. Fluorescence Spectra

Fluorescence spectra of the two SePEP and PEP samples (0.25 mg/mL in distilled water) were measured using an RF-6000 fluorescence spectrophotometer (Shimadzu Corporation, Kyoto, Japan). Distilled water was used as control. Emission spectra were collected with an excitation wavelength of 280 nm and an emission wavelength range of 320–500 nm at room temperature.

### 2.7. Fourier Transform Infrared (FTIR) Spectra

The FTIR spectra of the two SePEP and PEP samples were measured using a Nicolet iN10 MX Fourier Transform Microinfrared Spectrometer (Thermo Fisher Scientific, Waltham, MA, USA). The samples were mixed with spectroscopic-grade potassium bromide, ground, and pressed into pellets for analysis in the scanning range of 4000–400 cm^−1^.

### 2.8. Analysis of Amino Acid Composition

Amino acids were separated by cation exchange chromatography; ninhydrin reacted to give the corresponding derivatives, which were analyzed with an Evolution Pro UV-visible spectrophotometer (Thermo Fisher Scientific, Waltham, MA, USA), and the ninhydrin derivative of proline was detected at 440 nm, while the derivatives of the other 16 amino acids were measured at 570 nm [22].

Approximately 0.1 g of each of the two samples was placed in a hydrolysis tube, and 5 mL of 6 M hydrochloric acid were added. The tube was flushed with nitrogen gas, sealed, and placed in an oven at 110 °C for 22 h. The residue was dissolved with 100 mL of ultrapure water and mixed. A 1 mL aliquot was removed and dried under vacuum. Then, 1 mL of ultrapure water was added to redissolve the residue, mixed, and passed through a 0.22 μm aqueous filtration membrane. Then, 10 μL was placed in an L-8900 amino acid analyzer (Horiba Ltd., Kyoto, Japan) for sample determination. The column temperature was set at 55 °C, and the post-column reaction temperature was 135 °C.

### 2.9. Liquid Chromatography–Mass Spectrometry/Mass Spectrometry (LC-MS/MS)

#### 2.9.1. Sample Preparation

Samples’ preparation involved dissolving 1 mg of SePEPs or PEPs in 100 μL of 50 mM NH_4_HCO_3_ to obtain a concentration of 10 μg/μL. A 10 μL sample was mixed with 90 μL of 50 mM NH_4_HCO_3_ for further processing.

For reduction and alkylation, 1 μL of 1M DTT solution was added to each sample to reach a final concentration of 10 mM, followed by reduction in a 56 °C water bath for 1 h. Then, 2 μL of 1M iodoacetamide (IAM) solution was added to achieve a final concentration of 20 mM, and the reaction was allowed to proceed at room temperature in the dark for 40 min. Afterwards, 1 μL of 1M dithiothreitol (DTT) solution was added to neutralize unreacted IAM. C18 desalting columns (Thermo Fisher Scientific, Waltham, MA, USA) were used on each sample, followed by vacuum drying at 45 °C.

#### 2.9.2. Liquid Chromatographic Conditions

Peptide samples were separated using a C18 reverse-phase analytical column (75 μm i.d. × 50 cm, NanoViper, 2 μm particle size, 100 Å pore size). The mobile phases consisted of solvent A (0.1% formic acid in water) and solvent B (80% acetonitrile containing 0.1% formic acid). The flow rate was set to 400 nL/min, and the total run time was 65 min. The gradient elution program for solvent B was as follows: 1% at 0 min, increased to 6% at 4 min, 32% at 56 min, 99% at 60 min, and held at 99% until 65 min.

#### 2.9.3. Mass Spectrometry Conditions

With reference to Abid et al. [23], MS spectra (from *m*/*z* 350–2000) were acquired in the Orbitrap with resolution r = 60,000. The peptide fragmentation collision energy was 20 V.

### 2.10. Molecular Interaction Modeling Analysis

The BIOPEP database was used for online activity prediction of selenium-containing peptides. Based on the prediction results for SePEP and PEP samples, specific molecular interaction modeling was performed on the selected selenium-containing peptide. Peptides predicted to have DPPH activity were then docked using the Autodock Vina program [24]. Protein residues were visualized using the Pymol program, and the two-dimensional interactions between the receptor and ligand after docking were analyzed with the LigPlot program [24].

### 2.11. Peptide Synthesis

The peptide was synthesized using standard Fmoc-based solid-phase peptide synthesis (SPPS) on 2-chlorotrityl chloride (2-CTC) resin. The resin was accurately weighed and swollen in dichloromethane (DCM) for 1 h at room temperature, followed by three washes with N, N-dimethylformamide (DMF). The first Fmoc-protected amino acid (1 equiv.) and N, N-diisopropylethylamine (DIEA, 1.5 equiv.) were dissolved in DMF and added to the resin. The mixture was stirred at room temperature for 2 h to complete the initial coupling. After removal of the reaction solution, the resin was washed with DMF (3 × 5 mL). To cap any unreacted sites, a mixture of methanol and DIEA (*v*/*v* = 9:1) was added and reacted for 1 h at room temperature. The resin was then washed with DMF and subjected to Fmoc deprotection using 20% piperidine in DMF for 10 min, repeated twice. For the subsequent amino acid couplings, each Fmoc-protected amino acid (3 equiv.) was activated with 3 equiv. of 1-hydroxybenzotriazole (HOBt) and 3 equiv. of N, N′-diisopropylcarbodiimide (DIC) in DMF and reacted with the resin for 1.5 h at room temperature. The coupling and deprotection cycles were repeated sequentially for each residue until the full peptide sequence was assembled. After the final Fmoc deprotection, the resin was thoroughly washed with DMF and dried under vacuum. The cleavage of the peptide from the resin and side-chain deprotection was performed using a cleavage cocktail composed of trifluoroacetic acid (TFA), triisopropylsilane (TIS), 1,2-ethanedithiol (EDT), and water (95:2:2:1, *v*/*v*/*v*/*v*), incubated for 2 h at room temperature. The reaction mixture was filtered to remove the resin, and the filtrate was added dropwise into cold anhydrous diethyl ether to precipitate the crude peptide. The precipitate was collected by centrifugation at 4 °C (10,000 rpm, 10 min), washed twice with cold ether, and air-dried.

Purification of the crude peptide was carried out by reverse-phase high-performance liquid chromatography (RP-HPLC) on a SHIMADZU Inertsil ODS-SP C18 column (4.6 × 250 mm, 5 μm particle size). The mobile phase consisted of solvent A (0.1% TFA in water) and solvent B (0.1% TFA in acetonitrile). The flow rate was set to 1.0 mL/min, the column temperature was maintained at 25 °C, and detection was performed at 220 nm. An injection volume of 30 μL was used. Fractions corresponding to the major peak were collected, concentrated by rotary evaporation, and lyophilized at −80 °C for 24 h to obtain the final purified peptide.

### 2.12. DPPH Radical Scavenging Rates of Selenium-Containing Peptide

The DPPH radical scavenging activity of the selenium-containing peptide synthesized via solid-phase peptide synthesis (SPPS) was evaluated at concentrations ranging from 2 to 10 mg/mL. Glutathione (GSH) was used as a positive control.

### 2.13. Data Statistics and Analysis

Three independent experiments were conducted for each group. Statistical analyses were performed using SPSS 27.0 software (SPSS Inc., Chicago, IL, USA). The normality of the data was assessed using the Shapiro–Wilk test, and the homogeneity of variances was verified using Levene’s test. A one-way analysis of variance (ANOVA) was applied to evaluate differences among groups. When significant differences were observed, post hoc multiple comparisons were conducted using Tukey’s Honestly Significant Difference (HSD) test. Results are presented as mean ± standard deviation (SD), with statistical significance set at *p* < 0.05. Data visualization was carried out using Origin 2021 software (OriginLab Corp., Northampton, MA, USA).

## 3. Results

### 3.1. Comparison of In Vitro Antioxidant Activities of SePEPs and PEPs

**Figure 1:**
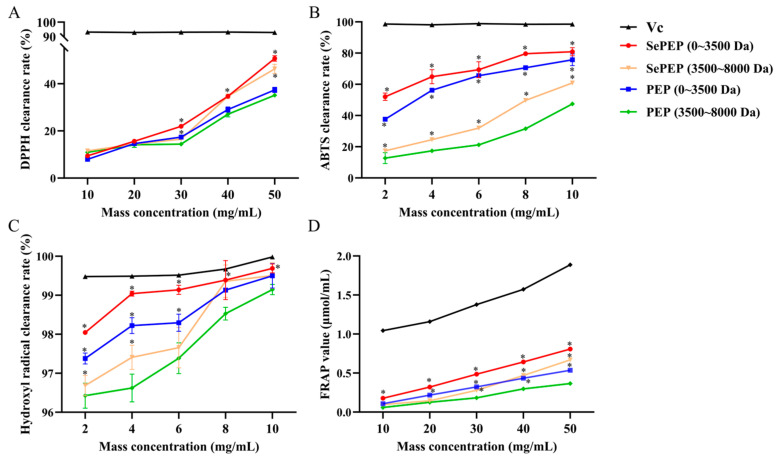
Antioxidant activities of SePEPs and PEPs. (**A**): DPPH radical scavenging rate; (**B**): ABTS radical scavenging rate; (**C**): hydroxyl radical scavenging rate; (**D**): FRAP total antioxidant capacity (* *p* < 0.05).

The antioxidant capacity increased with the increase in substrate peptide concentration (Figure 1). Among them, the SePEP fraction with a molecular weight of less than 3500 Da exhibited the strongest antioxidant activity, followed by the PEP fraction within the same molecular weight range. At a substrate concentration of 50 mg/mL, the DPPH radical scavenging rates for the 0–3500 Da SePEP and PEP fractions were 50.7 ± 1.2% and 37.4 ± 1.1%, respectively, and their FRAP values were 0.8 ± 0.02 µmol/mL and 0.5 ± 0.01 μmol/mL, respectively. At a substrate concentration of 10 mg/mL, the ABTS radical scavenging rates for the 0–3500 Da SePEP and PEP fractions were 80.9 ± 2.6% and 75.7 ± 3.8%, respectively, and the hydroxyl radical scavenging rates were 99.7 ± 0.1% and 99.5 ± 0.3%. Both organic selenium and peptides are bioactive components with antioxidant activity, and the chelation of selenium with peptides to form selenium-enriched peptides can enhance antioxidant activity [25,26]. The superior antioxidant capacity of SePEPs compared to PEPs is likely due to the combined antioxidant effects of selenium and peptides, where the synergy between selenium and polypeptides significantly boosts the overall antioxidant activity. Therefore, the SePEP and PEP fractions with molecular weights of 0–3500 Da were selected for subsequent structural identification.

### 3.2. TEM Analysis of SePEPs and PEPs

**Figure 2:**
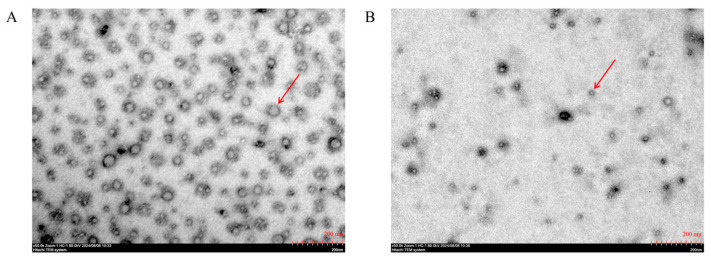
TEM of SePEPs (**A**) and PEPs (**B**). The red arrows denote peptide-related particles present in the field.

SePEPs and PEPs existed mostly in a single-molecule state (Figure 2), probably due to the disruption of the three-dimensional structure of the proteins by enzymatic reactions [13]. SePEPs were more tightly arranged and had a larger particle size than PEPs, probably because selenium bound to the peptide, which increased the particle size of SePEPs [27,28].

### 3.3. UV Absorption and Fluorescence Spectra

**Figure 3:**
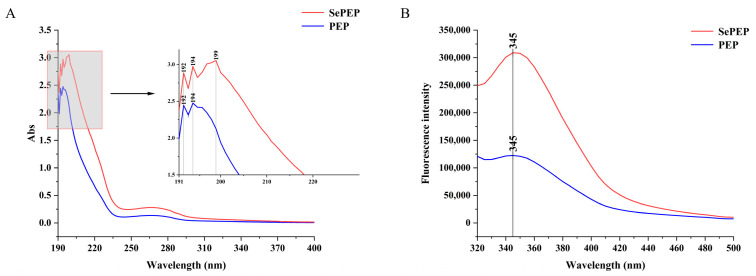
Absorption spectra (**A**) UV and (**B**) fluorescence of SePEPs and PEPs.

There were three absorption peaks for SePEPs and two absorption peaks for PEPs in the wavelength range of 190–400 nm (Figure 3A), which is consistent with the maximum absorption peaks of the peptides in the UV–visible spectrum [29]. Among them, SePEPs showed a greater absorption peak intensity than PEPs, which may be attributed to the existence of binding interactions between selenium and the peptide, resulting in a change in hydrophobicity. The generation of selenium-enriched peptides caused a change in the light absorption properties of the ligand, suggesting that valence electron leaps occurred in selenium binding to the peptide [30]. The change in the intensity of the absorption peak may be due to the chelation reaction between selenium and the peptide, and at the same time, the oxygen atom of the carbonyl group in the peptide may undergo a coordination reaction with selenium, which changes the structure of the peptide with a chromophore (C=O, –COOH) and a co-chromophore (C=O, –COOH), resulting in a change in the peak intensity [31]. In addition, SePEPs had an additional characteristic peak at 199 nm compared to PEPs, which may be due to the binding of selenium to the peptide, resulting in the presence of components such as selenite in the peptide bond, leading to the appearance of the characteristic peaks [32].

Both SePEPs and PEPs exhibited absorption peaks at 345 nm, with the absorption peak of SePEPs of greater intensity than that of PEPs (Figure 3B). The change in fluorescence intensity may be due to the chelation between selenium and the peptide [33]. Selenium may bind to the surface of the peptide, leading to a hypsochromic effect, which in turn causes changes in fluorescence intensity. Alternatively, the increase in fluorescence intensity may be attributed to the exposure of more aromatic amino acids at the binding site of selenium. Furthermore, selenium may facilitate energy transfer between amino acid residues, contributing to the enhanced fluorescence intensity observed at the absorption peak [34].

### 3.4. FTIR

**Figure 4:**
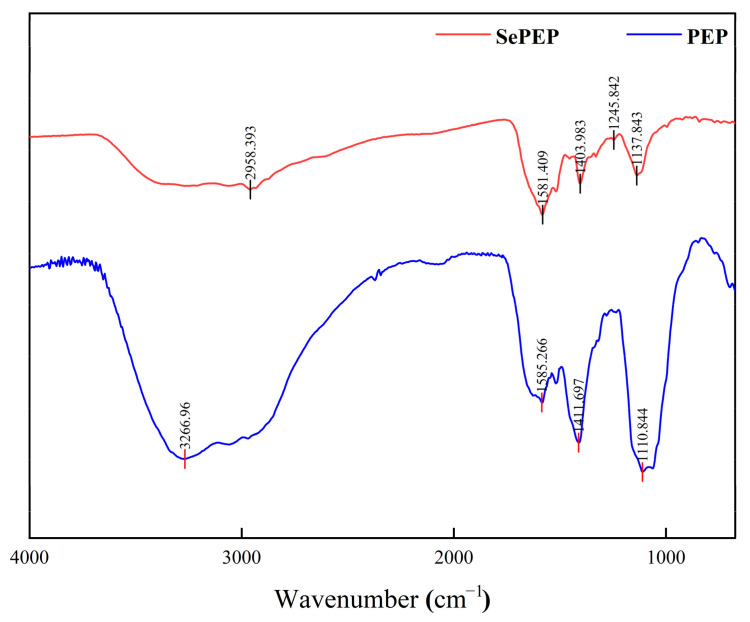
FTIR of SePEPs and PEPs.

Different chemical bonds and functional groups exhibit distinct vibrational absorption frequencies for infrared radiation, which are reflected as shifts in absorption peaks in the infrared spectrum; therefore, the position of absorption peaks in the infrared spectrum can be used to identify differences in the functional groups or chemical bonds between SePEPs and PEPs [35]. SePEPs and PEPs exhibited differences in their infrared spectra and peak positions (Figure 4), suggesting that selenium had undergone a chelation reaction with the peptide, resulting in changes to the molecular structure and the formation of new compounds [36]. In the Amide III region (1200–1300 cm^−1^), the peak in PEPs at 1111 cm^−1^, which is commonly attributed to C–N stretching coupled with N–H bending of the peptide backbone, underwent shifts in SePEPs. Specifically, these peaks moved to 1246 cm^−1^ and 1138 cm^−1^ in SePEPs, indicating modifications in the peptide backbone conformation upon selenium coordination [37]. This redshift, along with the broadening of the peaks, suggests that selenium binding influences the overall structure of the peptide. The peak at 1412 cm^−1^ in PEPs, associated with C–H bending, shifted to 1404 cm^−1^ in SePEPs. This subtle redshift further supports the idea that selenium binding alters the local vibrational environment of the methyl groups in the peptide chain [38]. Additionally, the amide II band at 1585 cm^−1^ in PEPs shifted slightly to 1581 cm^−1^ in SePEPs, likely due to changes in N–H bending and C–N stretching as a result of Se–N or Se–O bond formation. The sharp peak at 3267 cm^−1^ in PEPs, resulting from the stretching vibration of –OH or –NH groups, redshifted to 2958 cm^−1^ in SePEPs, suggesting the presence of hydrogen-bonding interactions between selenium and the hydroxyl or amino groups of the peptide [39]. Furthermore, in the region between 600 and 1000 cm^−1^, a series of weak absorption peaks in SePEPs are likely attributed to Se–O–C asynchronous stretching vibrations or Se–C vibrations (replacing the S–C bond in the peptide), further supporting the successful incorporation of selenium into the peptide structure [40].

### 3.5. Amino Acid Composition of SePEPs and PEPs

Amino acid composition plays a significant role in the antioxidant activity of functional peptides [40]. Studies have shown that higher levels of hydrophobic amino acids are associated with greater antioxidant activity [41]. SePEPs contained higher levels of total, essential, and hydrophobic amino acids compared to PEPs (Table 1), which contributed to the significantly enhanced antioxidant activity of SePEPs.

Previous studies have found that the amino acids chelated by selenium are mainly methionine (Met) and cysteine (Cys) [42]. Therefore, the content of Cys in SePEPs was lower than in PEPs, probably because selenium replaced the sulfur atom in Cys, resulting in a decrease [43]. Furthermore, the total amino acid and hydrophobic amino acid content in peptides are significantly correlated with antioxidant activity [41]. The higher content of hydrophobic amino acids in SePEPs further supported its superior antioxidant activity compared to PEPs. Additionally, the selenium atom may substitute certain amino acids on the peptide chain, leading to the formation of selenium-peptide bonds and altering the amino acid composition.

### 3.6. LC-MS/MS

**Figure 5:**
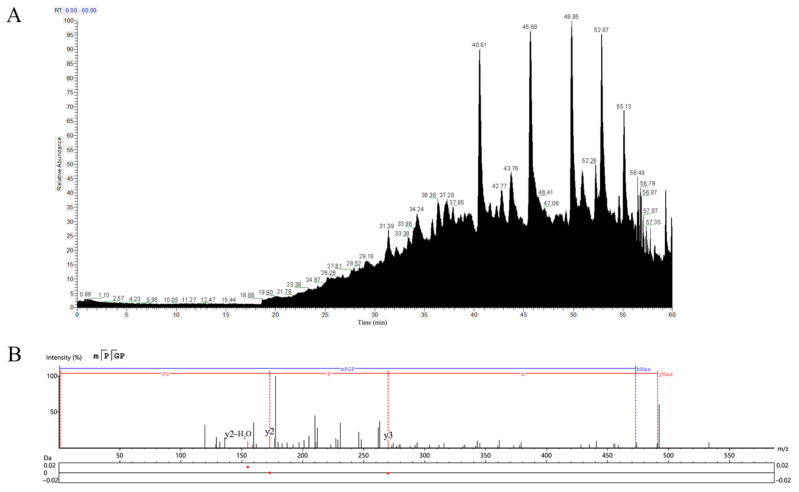
Total ion flow diagram of SePEPs (**A**) and secondary mass spectrum of MSePGP (**B**).

In the total ion flow diagram of SePEPs (Figure 5A), the retention time of SePEPs was mainly concentrated after 40 min. The molecular weight of selenium was found to be 47.94 Da higher than that of sulfur in peptide identification (Table 2). During the fragmentation ion matching process, methionine and cysteine, which had a molecular weight increase of 47.94 Da, were identified as selenomethionine and selenocysteine (Table 2). Based on the mass-to-charge ratio (*m*/*z*) in the MS/MS spectrum and the molecular weights of various amino acids, the peptide sequences corresponding to each MS/MS fragment were inferred. The peptide sequence represented in Figure 5B was identified as MSePGP. This sequence was then compared to the data of proteins of *P. eryngii*, and a 100% match was found. Using this method of sequence comparison, we analyzed the LC-MS/MS results, and 16 selenium-containing peptides were identified. The corresponding sequences of these peptides are listed in Table 2. The MS/MS spectra of the remaining identified selenium-containing peptides are provided in Supplementary Materials (Figure S1).

By comparing the selenium-containing peptides of SePEPs and PEPs, the results indicated that 16 selenium-containing peptides were identified from SePEPs. Among these, 13 were selenocysteine, and 3 were selenomethionine. Additionally, CSePY, CSeCPT, and FDGCSe were also identified in PEPs, so these three peptides were excluded from further analysis. Furthermore, we found that the selenium-containing peptides were mainly composed of three or four amino acids.

### 3.7. Molecular Virtual Screening of Selenium-Containing Peptides with DPPH

Antioxidant activity and bio-toxicity predictions of the selenium-containing peptides were conducted using the BIOPEP database, and it was found that three peptides (SCSePL, MSePGP, and CSeSPL) exhibited potential antioxidant activity, among which MSePGP showed no potential bio-toxicity (Table 3).

### 3.8. Molecular Interaction Modeling Visualization Analysis

**Figure 6:**
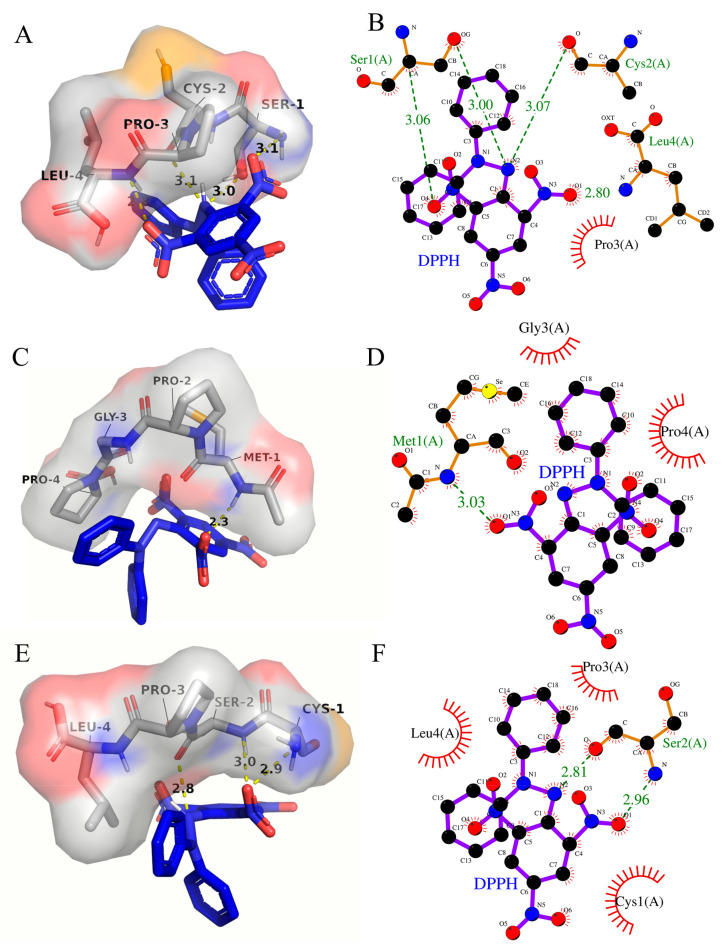
Interaction models between peptides containing selenium and DPPH ((**A**,**B**): SCSePL; (**C**,**D**): MSePGCP; (**E**,**F**): CSeSPL). Left column: 3D (the yellow regions represent the binding sites of selenium on the peptides); right column: 2D.

The binding energy between SCSePL and DPPH was −3.1 kcal/mol (Table 4). The amino acid residues involved in the interaction between DPPH and SCSePL were LEU-4, PRO-3, CYS-2, and SER-1 (Figure 6A,B). The N and O atoms on SER-1 formed a hydrogen bond with DPPH, with bond lengths of 3.06 Å and 3.00 Å, respectively. The O atom on CYS-2 formed a hydrogen bond with DPPH with a bond length of 3.07 Å. The N atom on LEU-4 formed a hydrogen bond with DPPH with a bond length of 2.80 Å. The PRO-3 residue stabilized the conformation through hydrophobic interactions. Thus, the interactions between SCSePL and DPPH were primarily driven by hydrogen bonding, with hydrophobic interactions playing a supplementary role. No hydrogen-bonding interaction was found between selenium and DPPH.

The binding energy between MSePGP and DPPH was −3.4 kcal/mol (Table 4). The amino acid residues involved in the interaction between DPPH and MSePGP were MET-1, PRO-2, GLY-3, and PRO-4 (Figure 6C,D). The N atom on Met-1 could form a hydrogen bond with the oxygen atom on DPPH, with a bond length of 3.03 Å. Additionally, the C-H bond on Met-1 formed a hydrogen bond with DPPH, with a bond length of 2.3 Å. Meanwhile, the GLY-3 and PRO-4 residues stabilized the structure through hydrophobic interactions, further stabilizing the ligand–ligand interaction.

The binding energy between CSeSPL and DPPH was −2.8 kcal/mol (Table 4). The amino acid residues involved in the interaction between DPPH and CSeSPL were LEU-4, PRO-3, CYS-1, and SER-2 (Figure 6E,F). The N and O atoms on SER-2 formed hydrogen bonds with DPPH, with bond lengths of 2.96 Å and 2.81 Å, respectively. The PRO-3, CYS-1, and LEU-4 residues stabilized the conformation through hydrophobic interactions.

The results indicated that the interactions between selenium-containing peptides (SCSePL, MSePGP, CSeSPL) and DPPH were mainly driven by hydrogen bonding, with hydrophobic interactions playing a supplementary role. This suggested that hydrogen-bonding interactions played a more significant role in stabilizing the structure. Among them, MSePGP had the shortest hydrogen bond length and the lowest binding energy, indicating the tightest and most stable binding between MSePGP and DPPH.

Moreover, although no direct hydrogen bonding was observed between selenium atoms and DPPH, the incorporation of selenium may influence the peptide’s conformation and local electrostatic environment, thereby indirectly enhancing its antioxidant activity. The redox properties of selenium are likely to play a key role in free radical scavenging. Selenocysteine, the predominant selenium-containing amino acid, possesses a lower redox potential than its sulfur-containing analog, cysteine, making it a more effective electron donor [44]. Therefore, the enhanced antioxidant activity of selenium-enriched peptides can be attributed to a synergistic mechanism involving hydrogen bonding, hydrophobic interactions, and the intrinsic redox potential of selenium.

### 3.9. DPPH Radical Scavenging Rates of the Selenium-Containing Peptide

**Figure 7:**
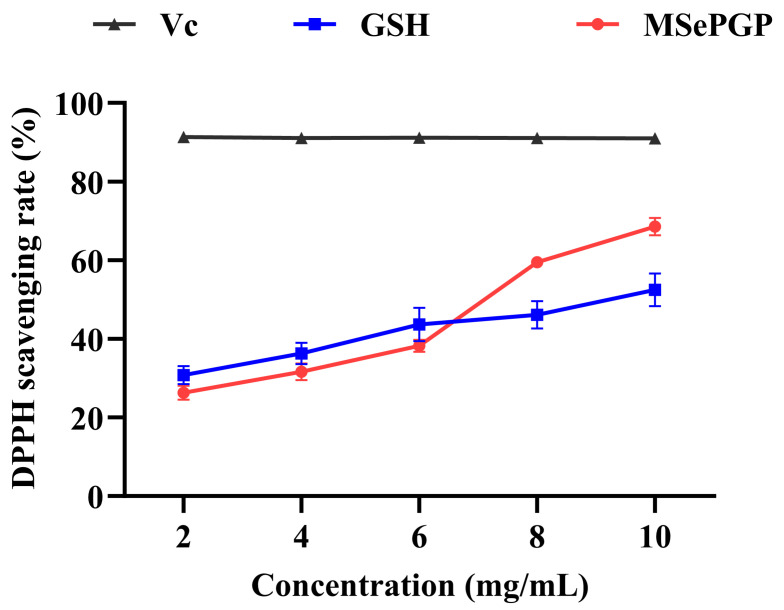
DPPH radical scavenging rates of the selenium-containing peptide (GSH = glutathione).

The selenium-containing peptide (MSePGP) identified in Section 3.8, which exhibited potential antioxidant activity and showed no potential bio-toxicity, was synthesized using solid-phase peptide synthesis to validate its DPPH antioxidant activity. The DPPH radical scavenging activity of MSePGP and glutathione (GSH) increased with the increase in substrate concentration (Figure 7). At lower concentrations (<6 mg/mL), the DPPH scavenging activity of GSH was higher than that of MSePGP. However, at higher concentrations (>8 mg/mL), MSePGP demonstrated significantly greater DPPH radical scavenging activity compared to GSH. At 10 mg/mL, the DPPH radical scavenging rate of MSePGP was 68.5 ± 2.2%, while that of glutathione was 52.5 ± 5.2%. These results indicated that MSePGP exhibited superior antioxidant activity over glutathione at higher concentrations.

Selenium is a key component of the active site of glutathione peroxidase (GPx) and is known to enhance antioxidant enzyme activity, thereby improving overall antioxidant capacity [45]. Additionally, PRO residues possess inherent antioxidant properties [46]. Therefore, at higher concentrations, the synergistic effects of selenium and amino acid residues in MSePGP may contribute to its enhanced free radical scavenging capacity.

## 4. Conclusions

In conclusion, selenium-enriched peptides (SePEPs) extracted from selenium-enriched *P. eryngii* exhibited notable antioxidant activity. During the biotransformation process, selenium replaced sulfur to form seleno-substituted amino acids, which were successfully identified by LC-MS/MS. These selenium-containing peptides demonstrated interactions with DPPH radicals, and the solid-phase synthesized peptides showed a certain degree of antioxidant potential. However, given the narrow safety margin of selenium intake in humans (approximately 0.4 mg per day), the development and application of selenium-containing peptides in food or health-related products should be approached with caution. Future research should not only focus on the prediction, design, and synthesis of antioxidant peptides, but also emphasize safety evaluation, dosage control, and bioavailability to ensure practical and safe use. This study provides a theoretical foundation for the further development of functional peptides with antioxidant properties.

## Figures and Tables

**Table 1:** Amino acid composition of SePEPs and PEPs.

Amino Acid	Amount (mg/g)	
	SePEPs	PEPs
Glutamic acid (Glu)	40.99	37.02
Leucine (Leu)	30.33	23.68
Alanine (Ala)	29.28	23.35
Aspartic acid (Asp)	25.92	21.18
Valine (Val)	23.64	17.78
Phenylalanine (Phe)	20.84	17.01
Proline (Pro)	15.85	8.68
Isoleucine (Ile)	15.51	13.07
Lysine (Lys)	14.89	13.44
Glycine (Gly)	13.98	14.91
Serine (Ser)	9.55	5.27
Histidine (His)	9.38	6.83
Threonine (Thr)	9.2	6.77
Tryptophan (Tyr)	8.44	7.26
Arginine (Arg)	8.15	5.10
Methionine (Met)	6.93	5.82
Cysteine (Cys)	0.9	1.59

**Table 2:** Identification results of selenium-containing peptides.

No.	Peptide	Mass (Da)	Length	ppm	*m*/*z*	z
1	CSeCL	427.068	3	−17.2	428.0679	1
2	CSeGF	415.065	3	−3.6	416.0704	1
3	FCSeH	453.092	3	7.5	454.1022	1
4	YGMSeT	518.128	4	9.6	519.1402	1
5	MSeCT	443.063	3	13.9	440.0764	1
6	CSeFA	429.080	3	−2.2	430.0866	1
7	CSeACL	498.105	4	−14.4	499.1052	1
8	SCSeSF	490.097	4	−4.9	491.1016	1
9	CSeAF	429.080	3	−1.9	430.0867	1
10	SCSePI	466.133	4	−13.9	467.1339	1
11	SCSePL	466.133	4	−13.9	467.1339	1
12	MSePGP	490.133	4	−10.5	491.1352	1
13	CSeSPL	466.133	4	−13.9	467.1339	1
14	CSePY	429.080	3	−4.2	430.0858	1
15	CSeCPT	512.084	4	−13.8	513.0846	1
16	FDGCSe	488.081	4	−3.2	489.0867	1

A = alanine, C = cysteine, F = phenylalanine, G = glycine, H = histidine, I = isoleucine, L = leucine, M = methionine, P = proline, S = serine, T = threonine, Y = tyrosine, D=Aspartic acid, Se=Selenium.

**Table 3:** Predictions of theoretical properties of selenium-containing peptides.

No.	Peptide	ABTS	DPPH	FRAP	ORCA	Bio-Toxicity
1	CSeCL	Active	Non-active	Non-active	Non-active	Active
2	CSeGF	Non-active	Non-active	Non-active	Non-active	Active
3	FCSeH	Non-active	Non-active	Non-active	Non-active	Active
4	YGMSeT	Non-active	Non-active	Non-active	Non-active	Non-active
5	MSeCT	Non-active	Non-active	Non-active	Non-active	Active
6	CSeFA	Non-active	Non-active	Non-active	Non-active	Active
7	CSeACL	Non-active	Non-active	Active	Non-active	Active
8	SCSeSF	Active	Non-active	Non-active	Non-active	Active
9	CSeAF	Non-active	Non-active	Non-active	Non-active	Active
10	SCSePI	Active	Non-active	Non-active	Non-active	Active
11	SCSePL	Active	Active	Active	Active	Active
12	MSePGP	Active	Active	Active	Active	Non-active
13	CSeSPL	Active	Active	Active	Active	Active

Se = selenium, ABTS = 2,2′-azinobis (3-ethylbenzothiazoline-6-sulphonic acid), DPPH = 1,1-diphenyl-2-picrylhydrazyl, FRAP = ferric reducing antioxidant power, and ORCA = oxygen radical absorbance capacity.

**Table 4:** The binding energy of selenium-containing peptides in DPPH docking.

No.	Name	Binding Energy
1	CCL	−3.0
2	CGF	−3.8
3	FCH	−3.9
4	YGMT	−3.7
5	MCT	−2.6
6	CFA	−3.4
7	CACL	−3.2
8	SCSF	−3.8
9	CAF	−4.0
10	SCPI	−3.2
11	SCPL	−3.1
12	MPGP	−3.4
13	CSPL	−2.8

## Data Availability

The original contributions presented in the study are included in the article; further inquiries can be directed to the corresponding author.

## Supplementary Materials

The following supporting information can be downloaded at: https://www.mdpi.com/article/10.3390/antiox14050586/s1. Figure S1: Secondary mass spectrum of CseCL; Figure S2: Secondary mass spectrum of CseGF; Figure S3: Secondary mass spectrum of FCSeH; Figure S4: Secondary mass spectrum of YGMSeT; Figure S5: Secondary mass spectrum of MSeCT; Figure S6: Secondary mass spectrum of CseFA; Figure S7: Secondary mass spectrum of CSeACL; Figure S8: Secondary mass spectrum of SCSeSF; Figure S9: Secondary mass spectrum of CSeAF; Figure S10: Secondary mass spectrum of SCSePI; Figure S11: Secondary mass spectrum of SCSePL; Figure S12: Secondary mass spectrum of CSeSPL.

## References

[1] Botha L.K., Namaumbo S., Kapito N.J., Ndovie P., Tsukuluza D.C., Jagot F., Mlangeni A.T. (2024). Selenium health impacts and sub-saharan regional nutritional challenges: A review. Res. Chem..

[2] Rashid M.T., Liu K., Ning M., Ullah K., Wali A., Jatoi M.A., Muzaffar N. (2024). Enhanced antioxidant activity of selenium-enriched brown rice protein against oxidative stress in mammalian erythrocytes under various cooking conditions. J. Agric. Food Res..

[3] Tutan D., Ulfberg J., Aydemir N., Eser B., Doğan İ. (2024). Selenium, a notable micronutrient: A crucial element in the context of all-cause long-term mortality in renal failure. Biol. Trace Elem. Res..

[4] Cai J., Su W., Chen X., Zheng H. (2022). Advances in the study of selenium and human intestinal bacteria. Front. Nutr..

[5] He Z., Gao J., Chen X., Ru Y., Zhang D., Pan X. (2025). Efficient recovery of heavy metals and selenium from wastewater using granular sludge: The crucial role of glutathione (GSH). Water Res..

[6] De Souza D.F., Da Silva M.D.S., De Souza T.C., Rocha G.C., Kasuya M.C.M., Eller M.R. (2023). Effect of selenium-enriched substrate on the chemical composition, mineral bioavailability, and yield of edible mushrooms. Biol. Trace Elem. Res..

[7] Dong Z., Xiao Y., Wu H. (2021). Selenium accumulation, speciation, and its effect on nutritive value of *Flammulina velutipes* (Golden needle mushroom). Food Chem..

[8] Yu A., Ji Y., Ma G., Xu J., Hu Q. (2023). Identification and preparation of selenium-containing peptides from selenium-enriched *Pleurotus eryngii* and their protective effect on lead-induced oxidative damage in NCTC1469 hepatocytes. J. Sci. Food Agric..

[9] Wang Y., Ji Y., Meng K., Zhang J., Zhong L., Zhan Q., Zhao L. (2024). Effects of different selenium biofortification methods on *Pleurotus eryngii* polysaccharides: Structural characteristics, antioxidant activity, and binding capacity in vitro. Int. J. Biol. Macromol..

[10] Wang B., Zhao N., Li J., Xu R., Wang T., Guo L., Ma M., Fan M., Wei X. (2021). Selenium-enriched *Lactobacillus plantarum* improves the antioxidant activity and flavor properties of fermented *Pleurotus eryngii*. Food Chem..

[11] Gąsecka M., Mleczek M., Siwulski M., Niedzielski P. (2016). Phenolic composition and antioxidant properties of *Pleurotus ostreatus* and *Pleurotus eryngii* enriched with selenium and zinc. Eur. Food Res. Technol..

[12] Wu L., Long L., Wen X., Qiu D., Yin H., Luo K. (2023). Study on enzymatic preparation and antioxidant activity of selenium-rich peptides from kidney bean leaves. Cereals Oils.

[13] Li L., Jin L., Guo P., Liu D., Fu J. (2024). Physicochemical properties, functional characteristics, and structure of selenium-enriched millet protein. Food Ferment. Ind..

[14] Yu T., Guo J., Zhu S., Li M., Zhu Z., Cheng S., Wang S., Sun Y., Cong X. (2020). Protective effects of selenium-enriched peptides from *Cardaminutese violifolia* against high-fat diet-induced obesity and its associated metabolic disorders in mice. Rsc Adv..

[15] Wu S., Wu Q., Wang J., Li Y., Chen B., Zhu Z., Huang R., Chen M., Huang A., Xie Y. (2022). Novel selenium peptides obtained from selenium-enriched *Cordyceps militaris* alleviate neuroinflammation and gut microbiota dysbacteriosis in LPS-injured mice. J. Agric. Food Chem..

[16] Hallin E.I., Serkland T.T., Bjånes T.K., Skrede S. (2024). High-throughput, low-cost quantification of 11 therapeutic antibodies using caprylic acid precipitation and LC-MS/MS. Anal. Chim. Acta.

[17] Olajide O.E., Zirpoli M., Kartowikromo K.Y., Zheng J., Hamid A.M. (2024). Discriminutesation of common *Escherichia coli* strains in urine by liquid chromatography-ion mobility-tandem mass spectrometry and machine learning. J. Am. Soc. Mass Spectra..

[18] Liu X., Liu K. (2024). Optimization of preparation process and activity of selenium-enriched peanut peptides with cholesterol-lowering activity. China Oils Fats.

[19] Han A., Liu H., Dai Y., Sun S., Ma H. (2024). Screening of umami peptides from fermented grains by machine learning, molecular docking, and molecular dynamics simulation. Food Biosci..

[20] Guo Y., Xu M., Hu X., Cen L., Pei D., Zhang D., Xu J., Shi P., Yang L., Cui H. (2024). Extraction, purification, and mechanism of immunomodulatory peptides obtained from silkworm pupa protein hydrolysate. Int. J. Biol. Macromol..

[21] Chen L., Wu H., Xu S., Zhu Z., Xie B., Chen B. (2025). Extraction process and antioxidant activities of selenopeptides from selenium-enriched *Pleurotus eryngii*. Mycosystema.

[22] Zhou X., Guo T., Lu Y., Hadiatullah H., Li P., Ding K., Zhao G. (2022). Effects of amino acid composition of yeast extract on the microbiota and aroma quality of fermented soy sauce. Food Chem..

[23] Abid K., Rochat B., Lassahn P.G., Stöcklin R., Michalet S., Brakch N., Aubert J.F., Vatansever B., Tella P., De Meester I. (2009). Kinetic study of neuropeptide Y (NPY) proteolysis in blood and identification of NPY3-35: A new peptide generated by plasma kallikrein. J. Biol. Chem..

[24] Nema R., Vats P., Singh J., Srivastava S.K., Kumar A. (2024). Competing endogenous TMPO-AS1-let-7c-5p- LDHA RNA network predicts the prognosis of lung adenocarcinoma patients. Asian Pac. J. Cancer Prev..

[25] Wu S., Zhu Z., Chen M., Huang A., Xie Y., Hu H., Zhang J., Wu Q., Wang J., Ding Y. (2023). Comparison of neuroprotection and regulating properties on gut microbiota between selenopeptide Val-Pro-Arg-Lys-Leu-SeMet and its native peptide Val-Pro-Arg-Lys-Leu-Met in vitro and in vivo. J. Agric. Food Chem..

[26] Zhao Q., McClements D.J., Li J., Chang C., Su Y., Gu L., Yang Y. (2024). Egg yolk selenopeptides: Preparation, characterization, and immunomodulatory activity. J. Agric. Food Chem..

[27] Zhu J., Du M., Wu M., Yue P., Yang X., Wei X., Wang Y. (2020). Preparation, physicochemical characterization, and identification of two novel mixed ACE-inhibiting peptides from two distinct tea alkali-soluble proteins. Eur. Food Res. Technol..

[28] Xiao Y., Cai W., Zheng Z., Ma H., Huang Q. (2020). Inhibition effect of *Lignosus Rhinocerotis* polysaccharides-selenium nanoparticles prepared by ultrasound treatment on non-enzymatic glycosylation. Food Sci..

[29] Zhang F., Zhang Y., Luo X., Liu P., Lin X., Deng Y., Jiang Y., Chen B. (2020). Purification and structural identification of polypeptides from fruiting bodies of *Volvariella volvacea*. Mycosystema.

[30] Wu J., Sun N., Lin S., Wu W. (2021). Preparation and structural characterization of peptide-selenium complex from hoki (*Macruronus novaezelandiae*) skin gelatin. Food Sci..

[31] Zhao L., Chen Z., Chen H., Hua P., Liu B. (2017). Optimization of chelation of juncao *Ganoderma lucidum* peptides with selenium by response surface methodology. Food Sci..

[32] Yang Z., Yin H., Wang J. (2023). Structural characterization and antioxidant activity in vitro of selenized *Bacillus subtilis* peptidoglycan. J. Henan Univ. Technol..

[33] Clarke S., Tamang S., Reiss P., Dahan M. (2011). A simple and general route for monofunctionalization of fluorescent and magnetic nanoparticles using peptides. Nanotechnology.

[34] Giordano A., Russo C., Raia C.A., Kuznetsova I.M., Stepanenko O.V., Turoverov K.K. (2004). Highly UV-absorbing complex in selenomethionine-substituted alcohol dehydrogenase from *Sulfolobus solfataricus*. J. Proteome Res..

[35] Borden J.T., Man A., Scott D.A., Liu K.Z. (2003). Tobacco-induced alterations to the Fourier-transform infrared spectrum of serum. J. Mol. Med..

[36] Jia J., Liu Q., Liu H., Yang C., Zhao Q., Xu Y., Wu W. (2024). Structure characterization and antioxidant activity of abalone visceral peptides-selenium in vitro. Food Chem..

[37] Hu Y., Lian W., Xu M., Yuan X. (2023). Preparation of selenium-polysaccharide from *Huaishan* yam and its inhibitory effect on α-glucosidase activity. J. Anhui Agric. Univ..

[38] Chen W., Yue L., Jiang Q., Xia W. (2019). Effect of chitosan with different molecular weights on the stability, antioxidant, and anticancer activities of well-dispersed selenium nanoparticles. IET Nanobiotechnol..

[39] Wang X., Fu J., Bhullar K.S., Chen B., Liu H., Zhang Y., Wang C., Liu C., Su D., Ma X. (2024). Identification, in silico selection, and mechanistic investigation of antioxidant peptides from corn gluten meal hydrolysate. Food Chem..

[40] Zhu L., Xie C., Su Y., Dong Y., Cheng S., He J., He Y. (2023). Process optimization, structural characterization, and antioxidant activities of black pigment extracted from Enshi selenium-enriched *Sesamum indicum* L.. LWT.

[41] Terriente-Palacios C., Rubiño S., Hortós M., Peteiro C., Castellari M. (2022). Taurine, homotaurine, GABA, and hydrophobic amino acids content influences “in vitro” antioxidant and SIRT1 modulation activities of enzymatic protein hydrolysates from algae. Sci. Rep..

[42] Huang Y., Yan S., Wang P., Fang W., Feng G., Bai Y., Xing X., Cai H. (2024). Selenium-chelating peptides from the hydrolysates of degreased antarctic krill (*Euphausia superba*) powder: Preparation, structural characterization, and antioxidant properties. Food Biosci..

[43] Zhu S., Li Y., Chen X., Zhu Z., Li S., Song J., Zheng Z., Cong X., Cheng S. (2024). Co-immobilization of Alcalase/Dispase for production of selenium-enriched peptide from *Cardaminutese violifolia*. Foods.

[44] Pálla T., Mirzahosseini A., Noszál B. (2020). Species-specific, pH-independent, standard redox potential of selenocysteine and selenocysteamine. Antioxidants.

[45] Ibrahim M., Muhammad N., Naeem M., Deobald A.M., Kamdem J.P., Rocha J.B.T. (2015). In vitro evaluation of glutathione peroxidase (GPx)-like activity and antioxidant properties of an organoselenium compound. Toxicol. Vitr..

[46] Khammuang S., Sarnthima R., Sanachai K. (2022). Purification and identification of novel antioxidant peptides from silkworm pupae (*Bombyx mori*) protein hydrolysate and molecular docking study. Biocatal. Agric. Biotechnol..

