# Supplementary material for: Structural Identification and Molecular Interaction Modeling Analysis of Antioxidant Activity Selenium-Enriched Peptides from Selenium-Enriched Pleurotus eryngii
Source: Antioxidants (Basel). 2025 May 13;14(5):586. doi: 10.3390/antiox14050586 (PMC12108474; doi:10.3390/antiox14050586)
Supplement: Supplementary file 1 [file antioxidants-14-00586-s001.zip › antioxidants-3578831-supplementary.pdf]

Supplementary Data

Attached below are the mass spectra of peptides derived from selenium-enriched *Pleurotus eryngii*.

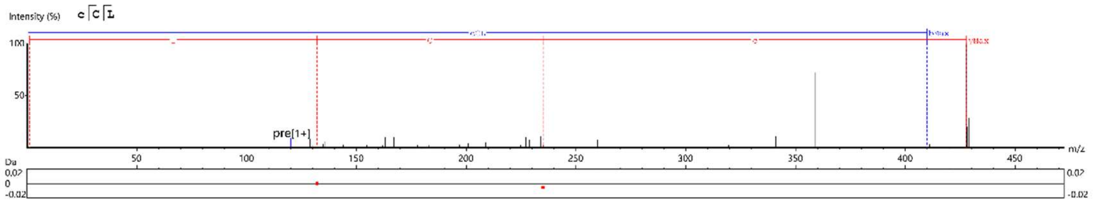

Figure S1. Secondary mass spectrum of CSeCL.

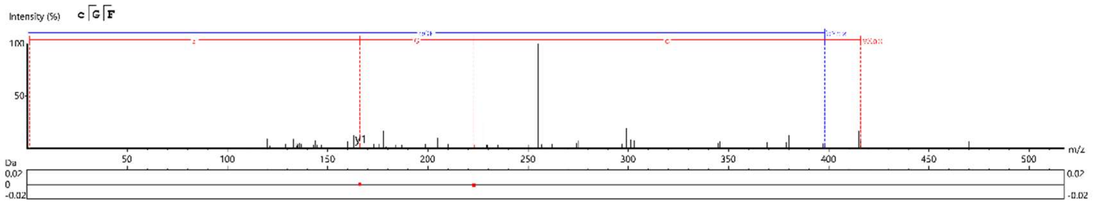

Figure S2. Secondary mass spectrum of CseGF.

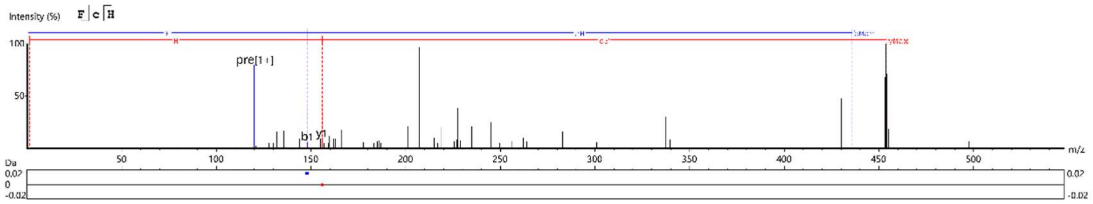

Figure S3. Secondary mass spectrum of FCSeH.

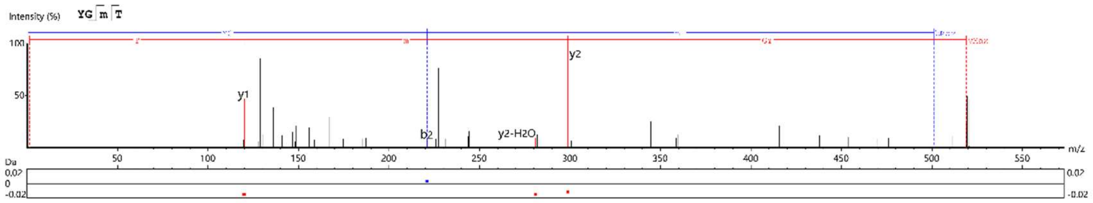

Figure S4. Secondary mass spectrum of YGMSeT.

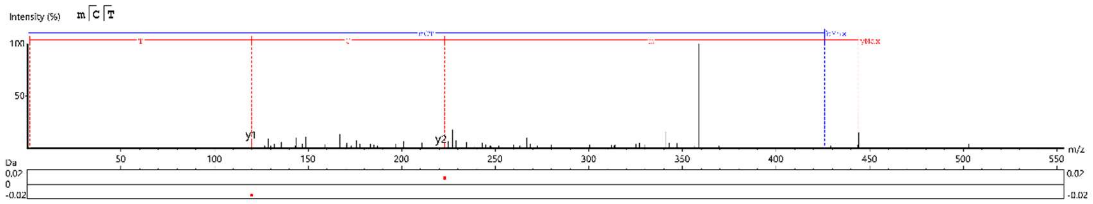

Figure S5. Secondary mass spectrum of MSeCT.

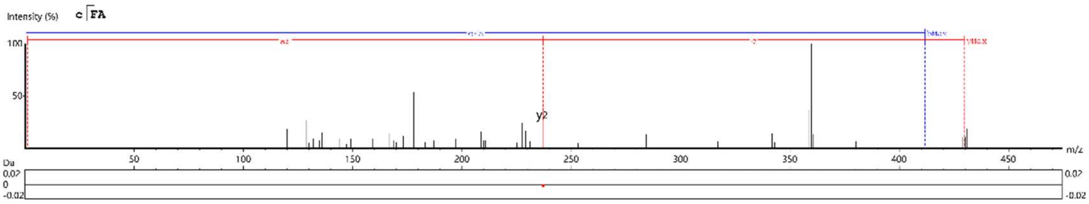

Figure S6. Secondary mass spectrum of CseFA.

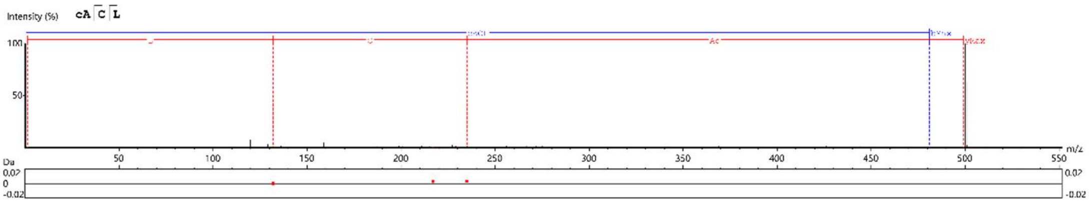

Figure S7. Secondary mass spectrum of CSeACL.

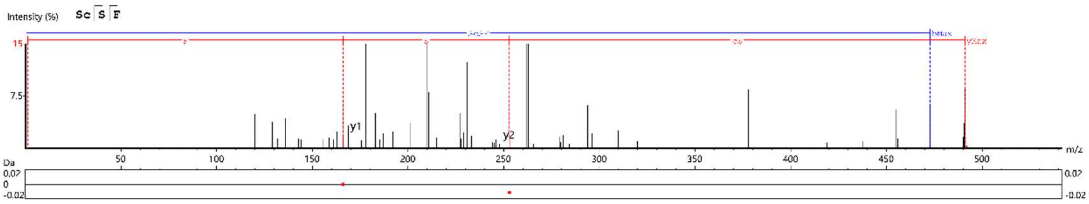

Figure S8. Secondary mass spectrum of SCSeSF.

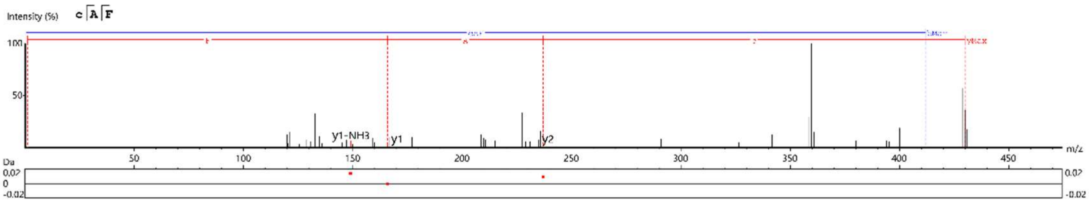

Figure S9. Secondary mass spectrum of CSeAF.

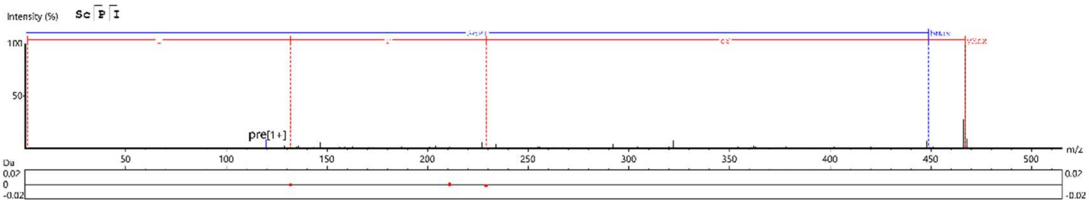

Figure S10. Secondary mass spectrum of SCSePI.

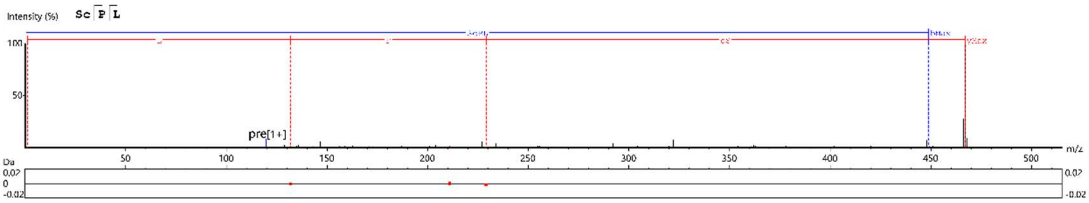

Figure S11. Secondary mass spectrum of SCSePL.

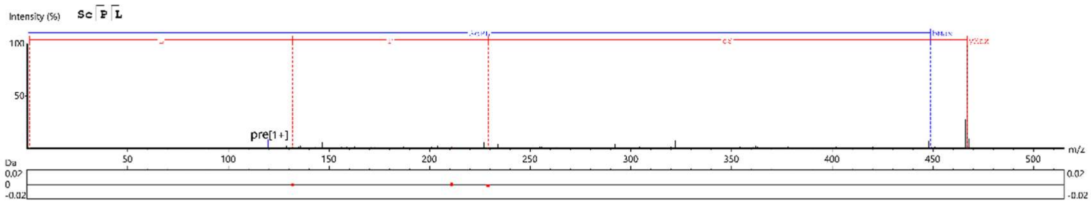

Figure S12. Secondary mass spectrum of CSeSPL.
